# A rare case of disseminated genitourinary tract tuberculosis complicated by emphysematous prostatitis and seminal vesicle abscess

**DOI:** 10.1259/bjrcr.20220101

**Published:** 2022-10-20

**Authors:** Preeya Kundasamy, Ben Kemp, Daniel Kearns, Andrew McCallum, Sarfraz Nazir, Paul C Lyon

**Affiliations:** 1 Department of Radiology, Oxford University Hospitals NHS Foundation Trust, Oxford, United Kingdom; 2 Department of Infectious Diseases, Oxford University Hospitals NHS Foundation Trust, Oxford, United Kingdom

## Abstract

Urogenital tuberculosis (UGTB) can affect the entire urinary tract including the kidneys, ureters (strictures), urinary bladder, prostate in addition to involving reproductive tracts. In modern day practice, both ultrasound and cross-sectional imaging play an important role in the radiological diagnosis of UGTB. The sequalae of untreated UGTB is morbid and can lead to end-stage renal failure, infertility, and life-threatening systemic infection. UGTB is less commonly observed in developed countries and may mimic other pathologies including malignancy. Thus, it is important that radiologists consider the differential diagnosis early, particularly individuals with risk factors such as travel to endemic regions, to allow optimal treatment and ensure best prognostic outcomes. UGTB can typically be managed by Infectious Disease clinicians with multidrug chemotherapy.

We have presented a case of microbiologically proven extrapulmonary tuberculosis (TB) predominantly involving the genitourinary tract. The response to TB agents and lack of evidence of co-infection with another organism, might suggest this as the first published case of emphysematous tuberculous prostatitis. Emphysematous prostatitis is indicative of a gas-forming infection of the prostate, and is associated with abscess formation in the vast majority of case and is an easily identified radiological feature on CT. It is not a well-recognised feature of *Mycobacterium tuberculosis* infection and thus microbiological diagnosis should be sought to confirm the diagnosis.

## Introduction

Tuberculosis (TB) is a multisystemic disease and remains the leading infectious cause of death worldwide. East Timor has a very high incidence of TB, with 508 cases/100,000 population reported in 2020 and the country has is an associated high mortality from TB.^1^ Oxfordshire has a sizeable East Timorese population with multiple health and social needs. Individuals born in East Timor form the largest group of new TB notifications in Oxfordshire [unpublished data].

Lymph node TB (LNTB) is the most frequent form of extrapulmonary TB. The genitourinary tract is another common site of extrapulmonary tuberculosis in the developing world (where over 90% of cases are seen)^2^ but affects just 1.5% of patients with tuberculosis overall. Urogenital tuberculosis (UGTB) is defined as infectious inflammation of urogenital system organs in any combination, caused by *Mycobacterium tuberculosis* complex.^3^ Dissemination from the primary focus of disease occurs via haematogenous, lymphatic or local spread from the principal focus of disease.^4^ Testicular tuberculosis is rare and occurs by spread from epididymoorchitis, while seminal vesicles are affected via retrograde canalicular transmission from the prostate or urinary tract.^5^ In females, the fallopian tubes, uterine endometrium and ovaries are affected. Signs and symptoms are non-specific including amenorrhea, adnexal masses and lymphadenopathy. Adhesions within the uterus (Asherman’s Syndrome) can lead to menstrual dysfunction and infertility.^6^ Case reports indicate that urogenital TB may be sexually transmitted.^7,8^

While the definite diagnosis of UGTB remains widely based on early morning urine cultures and histopathology, diagnosis is challenging and delayed, especially in low prevalence countries including the United Kingdom.^9–12^ The disease is frequently considered an imitator of other diseases such as urinary tract infection and malignancy, leading to incorrect or delayed diagnosis and subsequent complications such as infertility and renal failure. Therefore, non-specific urogenital presentations or features suggestive of malignancy amongst high-risk patients should prompt clinicians to maintain a high index of suspicion for UGTB.^12^

Tuberculous prostatitis has mainly been associated with the immunocompromised patient. In the context of immunosuppression, UGTB may manifest as sepsis, with bacteraemia and visceral metastatic foci.^13^ However, it can deceptively involve the prostate in immunocompetent individuals, mimicking cancer, emphasising the importance of including UGTB as a differential in high-risk patients.^14,15^ Tuberculous prostatitis is rare and the complication of prostatic abscess is extremely rare.^16^

Untreated UGTB has been previously shown to lead to ureteral strictures, contracted bladder, obstructive nephropathy, renal parenchymal infiltration and ultimately end stage renal failure.^17^ There is a case report of a patient presenting with emphysematous cystitis with pre-existing pulmonary TB, although the association is unclear and immunosuppressive status and diabetes may have been contributing factors.^18^ To date, there are no other published cases of emphysematous prostatitis due to TB.

UGTB is usually managed by Infectious Disease clinicians with multidrug chemotherapy, infrequent use of surgery and strict follow-up.^19^ In complex cases a multidisciplinary approach involving urology and/or radiological procedures may be required, *e.g.* for tissue diagnosis, debridement of necrotic tissue or abscess drainage.

## The role of imaging in diagnosis of UGTB

The value of cross-sectional imaging towards the evaluation of UGTB has been widely described and most recently proved pivotal towards raising suspicion of tuberculosis, although it has been highlighted that radiological appearances of UGTB are typically indistinguishable from those of other infectious causes.^20–22^ Recent literature highlights the key role of imaging as a method to assess the extent of disease involvement, identifying complications and guidance for interventional requirement such as biopsies or drainage.^22^

High resolution ultrasound has been described as the best imaging modality for assessing the epididymis, testis, scrotum and vas deferens to detect pathology such as inflammation, abscesses, large granulomas and calcification.^22^ Within the testes and epididymis, while some lesions demonstrate hyper or mixed echotexture, most of these lesions are hypoechoic.^23^ Occasionally, hypoechoic nodules are seen within the enlarged epididymis, displaying a miliary pattern. Progression of disease into the scrotal skin and tunica vaginalis may lead to hydrocele, pyocele, scrotal wall thickening and sinus formation.^22^ Tuberculous prostatitis is rare in the immunocompetent and may present with prostate gland enlargement with poorly defined hypoechoic lesions, usually in the posterolateral peripheral zones and irregularity of the external contour of the prostate.^24,25^ Colour doppler flow is increased in both tuberculous epidydimitis and prostatitis.^22^

Cross-sectional imaging (CT or MRI) has a significant role in identification of abscesses, lymphadenopathy, pulmonary involvement, musculoskeletal complications and soft tissue or parenchymal abnormalities in suspected cases of disseminated TB. Hydronephrosis can be severe when the renal pelvis and ureter are affected. Radiological CT features have included renal caliectasis, papillary necrosis and healing after the acute stage may include multifocal strictures. In established tuberculosis, the kidney atrophies and loses its structure, appearing as thin-walled cysts or as a multiloculated cyst.^20,26^ Chronic findings of untreated UGTB may include contracted urinary bladder.

Comparable to TB elsewhere, most cases demonstrate hypointense signal on *T*_2_ weighted images with regards to solid caseation, fibrosis and calcification on MRI. T2 hyperintensity has been observed with oedema or abscess formation.^27,28^ Prostate tuberculosis may appear as nodular and diffuse. In the former, the peripheral zone shows one or multiple T2-hypointense areas, whereas the diffuse pattern is typified by diffuse glandular enlargement. Abscesses are demonstrated as T2-hyperintense foci with central diffusion restriction and rim-enhancement.^22^

## Clinical course

A 30-year-old male, originally from East Timor who moved to UK 6 years previously, attended the Emergency Department with lower abdominal pain and dysuria. Urinalysis at that time demonstrated leucocytes and nitrites and he was treated for a lower urinary tract infection. 4 weeks later, he reattended the Emergency Department with left iliac fossa pain and dysuria for 3 days on a background of 4 weeks of increasing painful testicular swelling. Following discussion with the Urology team, the patient was referred for a testicular ultrasound to exclude testicular pathology including epididymitis. Ultrasound demonstrated a heterogenous lesion which appeared to be centred on the left epididymis but extending into the left testis. At this time, the differential diagnoses included infection (epididymo-orchitis) with a left testicular abscess, and the possibility of TB was raised, and a paratesticular tumour was deemed less likely. Tumour markers including alpha-feta protein (AFP) and human chorionic gonadotrophin (hCG) and circulating testosterone levels were all within normal limits.

The patient was discussed at the germ cell multidisciplinary team meeting where there was broad agreement with the initial differential diagnosis. TB was again considered as a possible causative agent. The patient was commenced on a 2-week course of (broad-spectrum) ciprofloxacin and doxycycline for an atypical orchitis, a regime which has potential for some anti-TB activity. Subsequent serial ultrasound scans demonstrated new lesions in the right testis and progression of the left testicular changes.

Subsequently, a contrast-enhanced CT was performed, with several salient imaging findings, which provided further evidence to support a radiological diagnosis of TB. The patient was referred on to the Infectious Diseases (ID) department for ongoing management. Microbiological confirmation was obtained by TB polymerase chain reaction (PCR) and culture of pus expressed from a scrotal sinus.

The patient had very mildly raised inflammatory markers throughout the clinical course with a C-Reactive Protein (CRP) of 21.6 (at presentation) and 17.8 mg/L (at the time of diagnosis). His full blood count (FBC) was normal other than a persistent, mild, eosinophilia (up to 1.02 × 10^9^/L). The eosinophilia was found to be the result of coinfection with *Strongyloides*, treated successfully with Ivermectin.

The patient was started on first-line quadruple TB therapy (rifampicin, isoniazid, pyrazinamide, ethambutol) with a significant reduction in his main symptom, scrotal swelling. Following a period where the patient returned to East Timor and was lost to earlier follow-up, the patient had a follow-up CT scan 8 months following the initial CT scan, with complete radiological resolution of the GU tract findings. The patient did not receive further treatment agents during this time.

## Imaging findings

**Figure 1.**
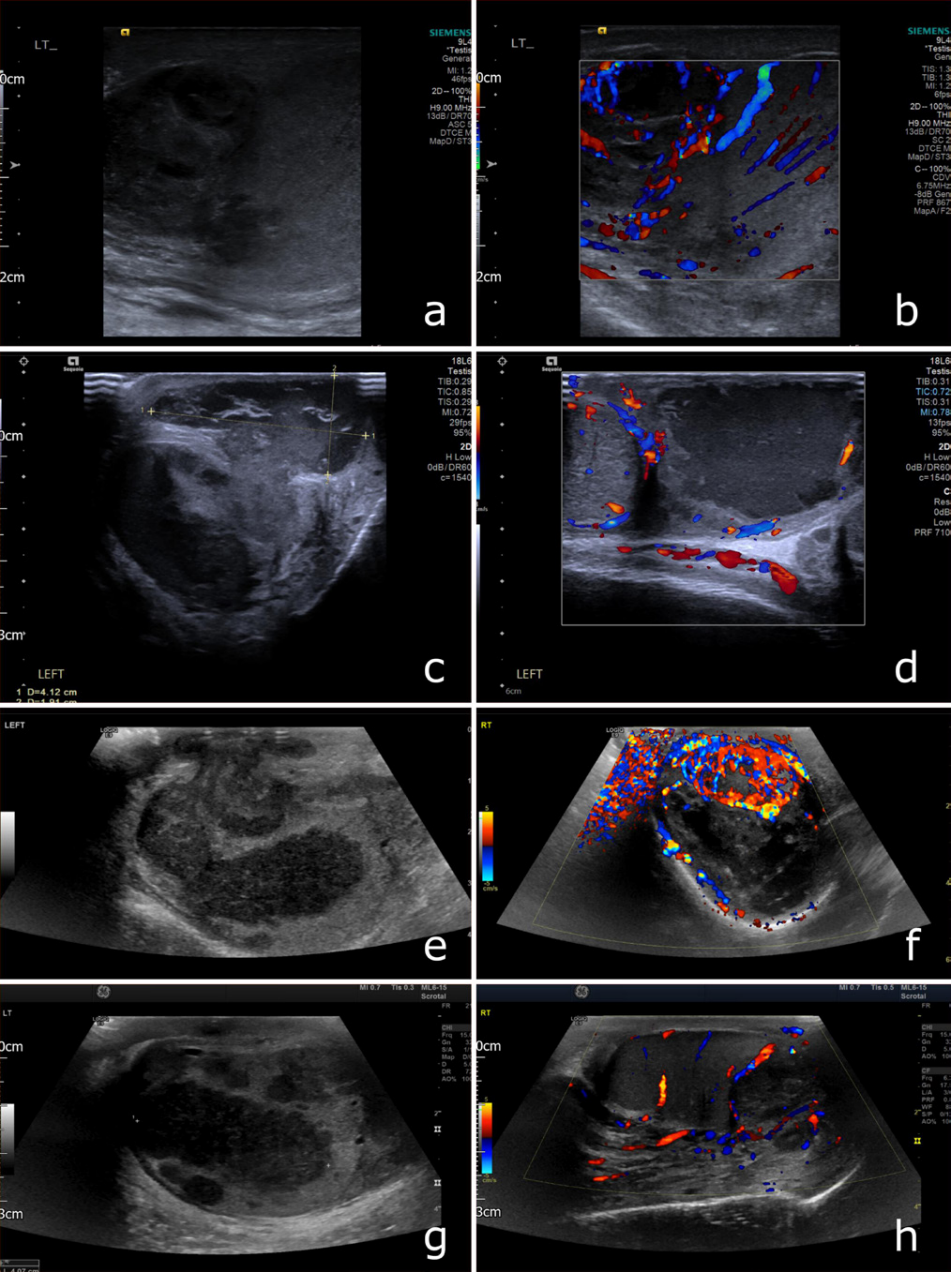
Ultrasound findings. (**a**) A large, heterogeneous, mass is seen centred on the head of the left epididymis at presentation. (**b**) The epididymal head and testis are hypervascular. (**c, d**) 3 weeks following the initial scan the lesion has progressed, extending further into the left testis; the right testis remains normal. (**e**) At 2 months, multifocal low attenuation lesions persist in the left testis. (**f**) There is also involvement of the right testis, with a floridly vascular lesion at the testicular hilum. (**g, h**) The testicular findings are similar at 3 months with less florid hypervascularity of the right testis. There is, however, new scrotal thickening (**g**) and discharge.

**Figure 2.**
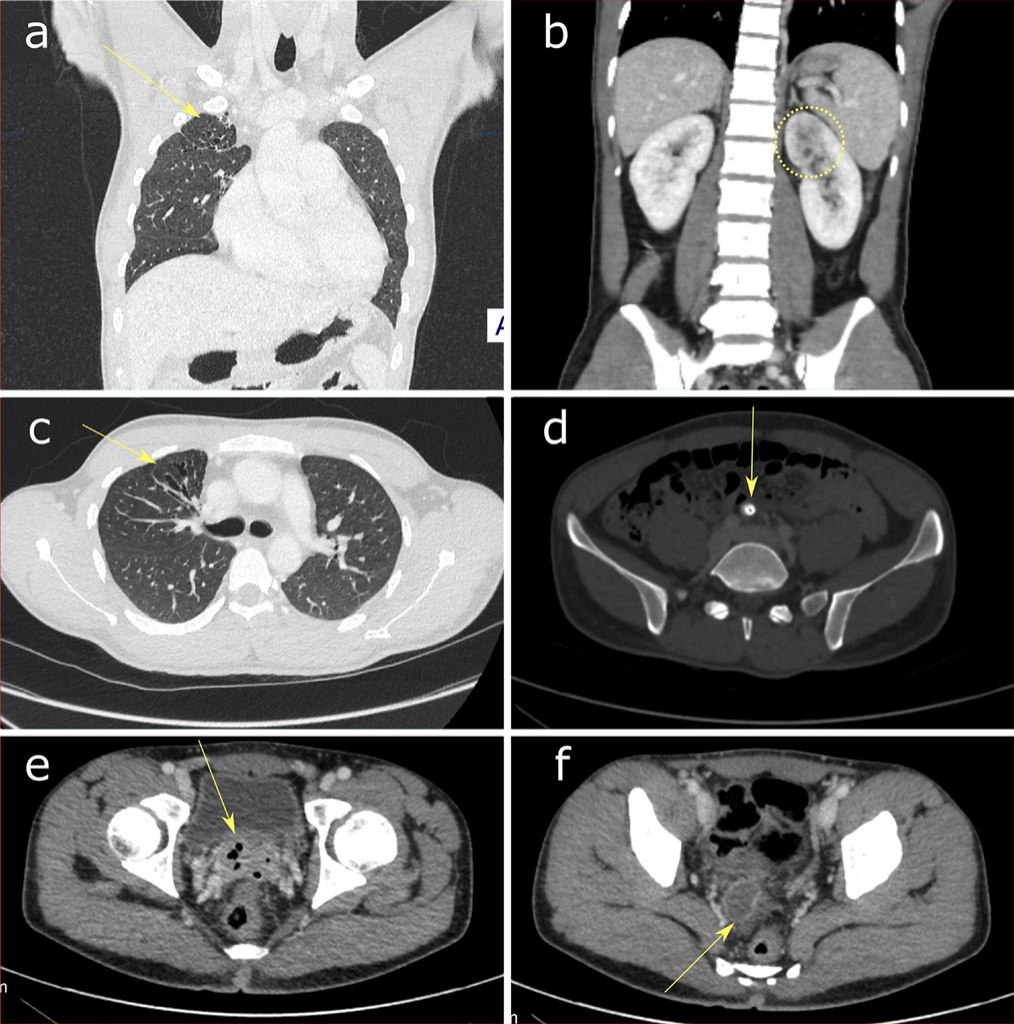
Contrast-enhanced CT Imaging. (**a, c**) Coronal and axial sections of the thorax in lung windows. There is right apical reticular change with pleuroparenchymal distortion and associated bronchiectasis (arrows). (**b**) Coronal view through the kidneys demonstrates an ill-defined left upper pole low attenuation hypoenhancement (dotted region), with normal enhancement of the remainder of the renal parenchyma bilaterally. (**d**) Axial view through the pelvis demonstrates a solitary peripherally calcified retroperitoneal lymph node (arrow). (**e**) Axial view through the deep pelvis demonstrates multiple small foci of gas within the prostate gland (arrow). (**f**) Axial view in the mid-pelvis demonstrates a peripherally enhancing low density collection in the region of the right seminal vesicles.

Serial testicular ultrasound scans revealed progressive epididymis and testicular hypoechoic lesions, in keeping with TB abscesses, initially confined to the left epididymis, subsequently progressing to left testis and then bilaterally with scrotal thickening (Figure 1). Whilst the ultrasound imaging was suggestive of TB, it was not diagnostic and microbiological correlation was required. Contrast- enhanced CT imaging of the chest, abdomen and pelvis was obtained 1 day before the final ultrasound images (g) and (h) of Figure 1. The CT revealed features of reactivated TB (Figure 2). Specifically, the chest CT demonstrates a focal region of pleuroparenchymal distortion and associated bronchiectasis in the right apex (Figure 2(a),(c)). A solitary peripherally calcified retroperitoneal lymph node is demonstrated within the pelvis (Figure 2(d)). Both findings are strongly suggestive of sequelae from previous pulmonary TB infection in this clinical context. There was no evidence of active pulmonary TB on the CT chest and no thoracic lymphadenopathy was demonstrated.

The CT abdomen and pelvis demonstrates hypoperfusion in the upper pole of the left kidney without hydronephrosis, consistent with direct infection of the renal parenchyma with TB. The remainder of the renal parenchyma was normally enhancing (Figure 2(b)).

Correlating with the ultrasound findings (Figure 1), there is a peripherally enhancing low density collection in the mid-pelvis which represents a TB abscess of the right seminal vesicle (Figure 2(f)). Furthermore, in the deep pelvis there are multiple small foci of gas within the prostate gland, consistent with emphysematous prostatitis (Figure 2(e)), with no radiological evidence of rectal fistula.

## Microbiological findings

Microbiological confirmation was obtained from pus expressed from a sinus on the scrotum. Rapid diagnosis was obtained by Cepheid MTB/RIF TB PCR: this confirmed the presence of *Mycobacterium tuberculosis (Mtb*) complex with no molecular markers for rifampicin resistance. Pus was cultured in Mycobacterial Growth Indicator Tubes and positive growth was confirmed as *Mtb* by lateral flow. Whole genome sequencing at the National Mycobacterium Reference Service (South) confirmed the presence of *Mtb* and susceptibility to first-line anti-tuberculosis therapy and quinolones.^29^ There was no evidence for co-infection with another organism, although deeper (prostatic) sampling was not performed.

## Case summary

We have presented a case of microbiologically proven extrapulmonary TB predominantly involving the genitourinary tract, including emphysematous tuberculous prostatitis, which is an uncommon clinical finding and may be neglected as a radiological diagnosis. Emphysematous prostatitis is an easily identified radiological feature on CT imaging. It is indicative of a gas-forming infection of the prostate, and which is associated with abscess formation in the vast majority of cases. It is not be a well-recognised feature of *Mtb* infection and thus microbiological diagnosis should be sought to confirm the diagnosis. The response to TB agents and lack of evidence of co-infection with another organism might suggest this as the first published case of emphysematous prostatitis due to TB.

## Learning points

- UGTB remains rare in developed countries
- UGTB an often presents with non-specific symptoms and can mimic other pathologies such as renal tract malignancy
- Early radiological recognition of UGTB is key to early diagnosis and improved outcomes
- It is important to have a high-index of suspicion for UGTB in patients from endemic regions
- UGTB is typically managed by Infectious Disease clinicians with multidrug chemotherapy
- A multidisciplinary approach involving urology and/or radiological procedures may be required in complex cases
- Strict follow-up following treatment is crucial given the often indolent nature of systemic TB
- Tuberculous prostatitis is rare and the complication of prostatic abscess is extremely rare
- We believe this may be the first reported case of emphysematous tuberculous prostatitis.
